# Arabidopsis Leaf Chloroplasts Have a Specific Sphingolipidome

**DOI:** 10.3390/plants13020299

**Published:** 2024-01-19

**Authors:** Chang Yang, Ling-Yan Wang, Yong-Kang Li, Jia-Ting Lin, Ding-Kang Chen, Nan Yao

**Affiliations:** State Key Laboratory of Biocontrol, Guangdong Provincial Key Laboratory of Plant Resources, School of Life Sciences, Sun Yat-sen University, Guangzhou 510275, China; yangch323@mail2.sysu.edu.cn (C.Y.); wangly6@mail3.sysu.edu.cn (L.-Y.W.); linjt36@mail2.sysu.edu.cn (J.-T.L.); cdkon@outlook.com (D.-K.C.)

**Keywords:** *Arabidopsis thaliana*, chloroplast, sphingolipidome, LC-MS/MS

## Abstract

Sphingolipids are ubiquitous in eukaryotes and certain prokaryotes, where they serve as vital components of biological membranes and bioactive molecules. Chloroplasts have complex membrane structures that play crucial roles in photosynthesis, but their specific sphingolipidome remains unreported. In this study, we used liquid chromatography–mass spectrometry (LC-MS/MS) to analyze the sphingolipidome of purified *Arabidopsis thaliana* chloroplasts. We detected 92 chloroplast sphingolipids. The chloroplast sphingolipidome differed from total leaf (TL) samples, with a higher content of free long-chain bases and hydroxyceramides and a greater proportion of complex sphingolipids with 16C fatty acid (FA) forms. Notably, chloroplast glucosylceramides were predominantly the d18:1 h16:0 and t18:1 h16:0 forms rather than the 24C FA form found in TL and other cellular structures. Comparing the sphingolipidomes of different cellular structures underscores the inhomogeneity of the intracellular distribution of sphingolipids. This provides a robust reference for further elucidating the function of sphingolipids in plant cells.

## 1. Introduction

Sphingolipids are ubiquitous in eukaryotes and certain prokaryotes that are composed of long-chain base (LCB) and fatty acid (FA) chains [1,2]. They are essential constituents of biological membranes, where they maintain the membrane’s structural integrity and biophysical properties [3,4]. Moreover, they serve as bioactive molecules, playing pivotal roles in plant growth, development, and responses to biotic and abiotic stresses. These functions include the regulation of cell death, organ development, resistance to abiotic stresses (salt, drought, and cold), defense against pathogens, and the promotion of rhizobial symbiosis [5,6,7,8,9,10].

The structural diversity of sphingolipids results from the variation in LCB species, FA chain lengths, hydroxylation, unsaturation, and modifications of the head group [2]. The LCB can be conjugated with FA chains and polar head groups to form complex sphingolipids. The common complex sphingolipids in plant cells include ceramide (Cer), hydroxyceramide (hCer), glucosylceramide (GlcCer), and glycosylinositolphosphoceramide (GIPC), with GIPC being the most abundant sphingolipid in plant cells [11].

The majority of enzymes involved in sphingolipid biosynthesis and metabolism localize in the endoplasmic reticulum (ER) and Golgi apparatus, and their products are subsequently transferred and transported to other cellular locations [12,13]. The advent of liquid chromatography–mass spectrometry (LC-MS/MS) technology has facilitated the high-resolution identification of plant sphingolipids [14]. Studies have provided detailed descriptions of the sphingolipidome at the level of the plant strain and organ, as well as its dynamic changes under varying conditions [15,16].

Sphingolipids are not uniformly distributed in cellular membrane systems [13]. However, our understanding of the sphingolipidome in various membrane-containing organelles of plants remains incomplete. Previous studies have reported that GlcCer and GIPC constitute 40% of the plasma membrane (PM) lipids [17,18], and sphingolipids account for approximately 20% of the lipids in ER and Golgi membranes, playing a crucial role in maintaining their morphology and function [19,20,21,22]. A recent study determined that the sphingolipid composition of vacuolar membranes (VM) differs from that of the PM, detergent-resistant membrane (DRM), and microsomal membrane (MIC), with a higher proportion of GlcCer rather than GIPC [23]. Additionally, the sphingolipid composition of plant mitochondria (Mito) has recently been reported to differ from that of other organelles and membrane systems [24]. These findings suggest that plant organelles have distinct sphingolipidomes.

Chloroplasts and their thylakoids constitute the most expansive membrane system in leaf mesophyll cells and are abundant in lipids [25]. Additionally, a rigorously regulated mechanism traffics lipids between the outer membrane of the chloroplast and the ER, underscoring the complexity and significance of lipid dynamics within plant cells [26]. However, the sphingolipidome of chloroplasts has yet to be reported. In this study, we delineate the sphingolipidome of chloroplasts in Arabidopsis using LC-MS/MS. We illuminate the unique characteristics of the sphingolipid composition within the chloroplast and show that it can be distinguished from that of the total leaf or other organelles.

## 2. Results

### 2.1. Purification of Chloroplast Fractions

**Figure 1:**
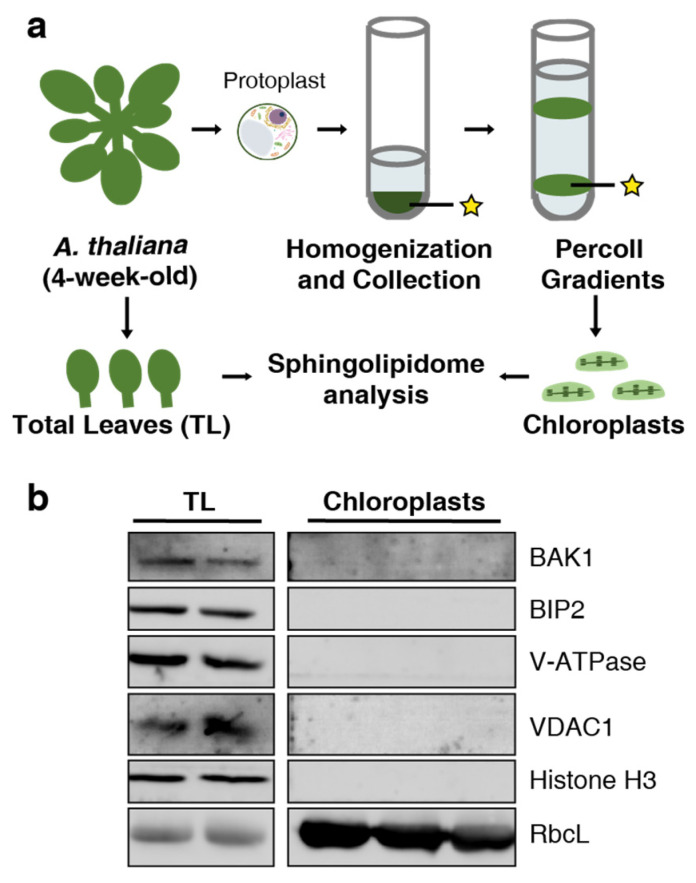
Experimental procedure for the sphingolipid analysis and purity assessment. (**a**) An experimental approach to obtain total leaf (TL) and chloroplast samples from Arabidopsis plants for sphingolipid analysis. Yellow asterisks indicate a target precipitation containing chloroplasts. (**b**) Immunodetection of BAK1 from the plasma membrane, BIP2 from the endoplasmic reticulum, RbcL from chloroplasts, V-ATPase from vacuoles, VDAC1 from the mitochondria, and histone H3 from nuclei. Proteins were separated using SDS-PAGE and detected via immunoblot. At least two biological replicates were performed for immunodetection with all protein markers. RbcL was used as a loading control.

In this study, we characterized the sphingolipid composition of the total leaf (TL) and chloroplast samples. The sphingolipidome of the total leaf has been widely reported [14]. We, therefore, used total leaf sphingolipid data as controls to compare against chloroplasts. The procedure for chloroplast isolation, which has been simplified for the purposes of this experiment, is depicted in Figure 1a. To assess the purity of the isolated chloroplast fraction, we employed immunoblotting assays using several marker proteins. These were as follows: BRASSINOSTEROID INSENSITIVE 1-ASSOCIATED RECEPTOR KINASE 1 (BAK1) for the plasma membrane, luminal BINDING PROTEIN 2 (BIP2) for the endoplasmic reticulum (ER), the Rubisco large subunit (RbcL) for the chloroplast, H(+)-ATPASE for the vacuole (V-ATPase), VOLTAGE-DEPENDENT ANION CHANNEL 1 (VDAC1) for the mitochondrion, and histone H3 for the nucleus. All these marker proteins were detectable in the TL samples. However, in the chloroplast samples, only RbcL was detected, and it was found to be enriched (Figure 1b). These findings suggest that the chloroplast samples used in this study were of high purity.

### 2.2. Overall Assessment of Sphingolipid Profiles

**Figure 2:**
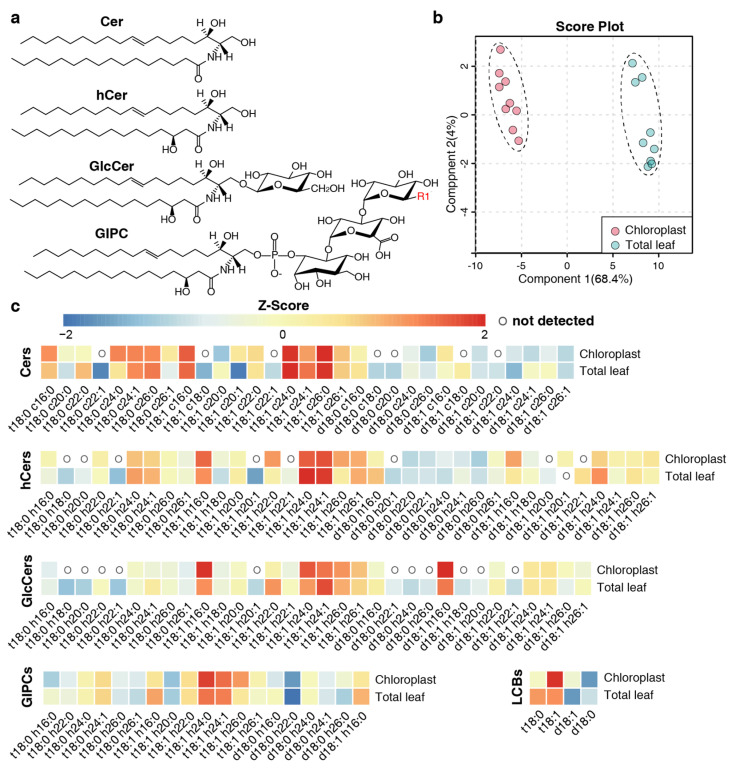
Multivariate analysis and heatmap plots of sphingolipid profiles. (**a**) Representation of the structure of main complex sphingolipid classes. The R1 in the structure of GIPC indicates mannose, glucosamine, N-acetyglucosamine, galactose, arabinose, or a combination of these hexose groups as described in [27]. (**b**) Partial least squares-discriminant analysis (PLS–DA) of sphingolipid profiles of the chloroplast and total leaf samples. The analysis is based on the relative content (mol %) of all detected sphingolipid species. In this study, nine individual plants were set for each sample type. (**c**) Heatmap plots of the abundance of LCBs, Cers, hCers, GlcCers, and GIPCs. The Z-score represents the standardized content of individual sphingolipid species in mol %. The scale from the left value to the right value represents the number of standard deviations from the average of each row (i.e., sample types), with red indicating higher contents than average and the blue color indicating lower contents than average. An “O” in the heatmap means that sphingolipid species were not detected in this sample group. The sphingolipid species that were not detected in any of the samples are not shown.

The sphingolipid profiles, encompassing LCBs and complex sphingolipids (Cers, hCers, GlcCers, and GIPCs) (Figure 2a), were comprehensively analyzed using LC-MS/MS. To ensure the reliability of the results, components exhibiting irregular peak shapes or low-peak values were deemed to be undetected. We identified 121 and 96 sphingolipid species in the TL and chloroplast samples, respectively (Figure 2c, Supplementary Tables S1 and S2). Specifically, four LCB species (d18:0, d18:1, t18:0, and t18:1) were universally detected across all samples (Figure 2c). In the chloroplast samples, 24 Cers, 28 hCers, 21 GlcCers, and 19 GIPC species were detected, and in the TL samples, 31 Cers, 35 hCers, 32 GlcCers, and 19 GIPC species were detected. Partial least squares–discriminant analysis (PLS–DA) indicated differences in the sphingolipidome in the two different sample types (Figure 2b).

### 2.3. Distribution of Sphingolipid Classes

**Figure 3:**
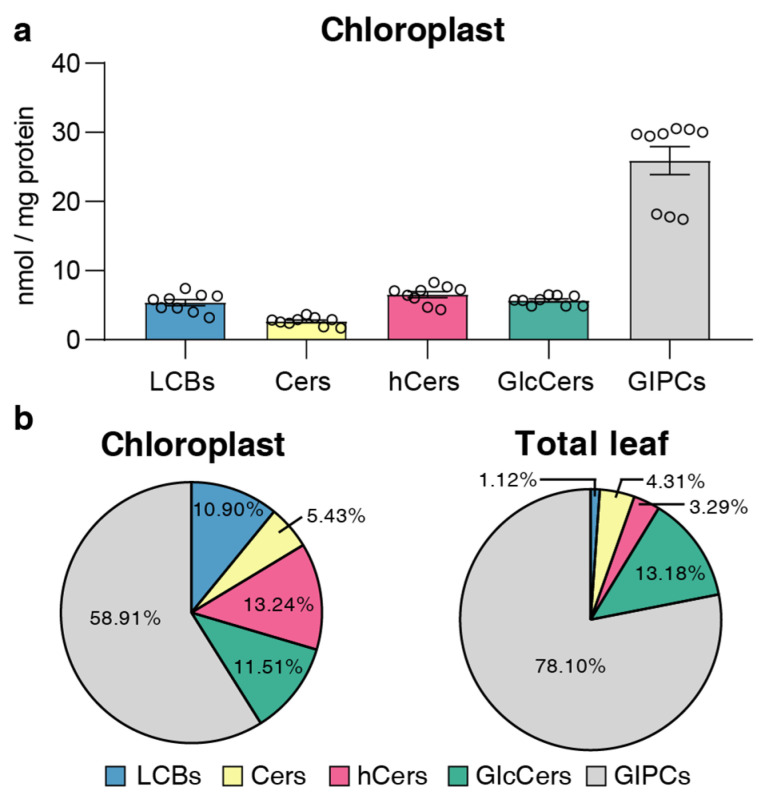
Content and distribution of sphingolipid classes. (**a**) Absolute content of five sphingolipid classes (LCBs, Cers, hCers, GlcCers, and GIPCs). The sphingolipid contents were expressed per mg of the protein in chloroplasts. Error bars represent the means ± SE from nine individual plants. (**b**) Distribution of sphingolipid classes. Percentage values in pie charts represent the average relative content of each sphingolipid class in chloroplast and total leaf samples.

The numbers of five sphingolipid classes (LCBs, Cers, hCers, GlcCers, and GIPCs) were expressed in terms of the absolute content (per mg protein of chloroplasts and per g dry weight (DW) of leaves) (Supplementary Table S1) and relative content (molecular percentage, mol %) (Supplementary Table S2). In terms of absolute sphingolipid content, LCBs, Cers, hCers, and GlcCers in chloroplasts were present in similar amounts, ranging from 1.5 to 10 nmol/mg protein, while GIPCs were present in significantly higher amounts of approximately 30 nmol/mg protein (Figure 3a).

We used relative content (mol %) for all the next comparisons to ensure that the data of chloroplast and TL sphingolipids were the same condition (Supplementary Table S2). This revealed notable differences between the chloroplast and TL samples (Figure 3b). GIPCs were overwhelmingly predominant in both chloroplast and TL samples, constituting about 60% to 80% of the total sphingolipid content. GlcCers were equally present in all samples, from 10% to 14%. In TL samples, LCBs constituted only about 1% of the total content, while in chloroplast samples, they accounted for a larger proportion of about 10%. Similarly, the proportion of Cers and hCers in TL samples was low (3% to 5%), while in chloroplast samples, the proportion of hCers was about 11%. These findings suggest a different distribution of sphingolipid classes between the chloroplasts and total leaf samples.

### 2.4. LCB and FA Profiling of Sphingolipids

In the case of free LCBs, all samples were predominantly composed of trihydroxylated forms (t18:0 and t18:1) as opposed to dihydroxylated forms (d18:0 and d18:1). The unsaturated LCBs (t18:1 and d18:1), which were desaturated at the C-8 position [28], were more abundant than their saturated counterparts. In chloroplast samples, t18:1 was overwhelmingly dominant, accounting for 70% to 90% of the total content in mol %. However, in TL samples, the proportion of t18:1 was approximately 40%, which is similar to that of t18:0 (Figure 2c).

**Figure 4:**
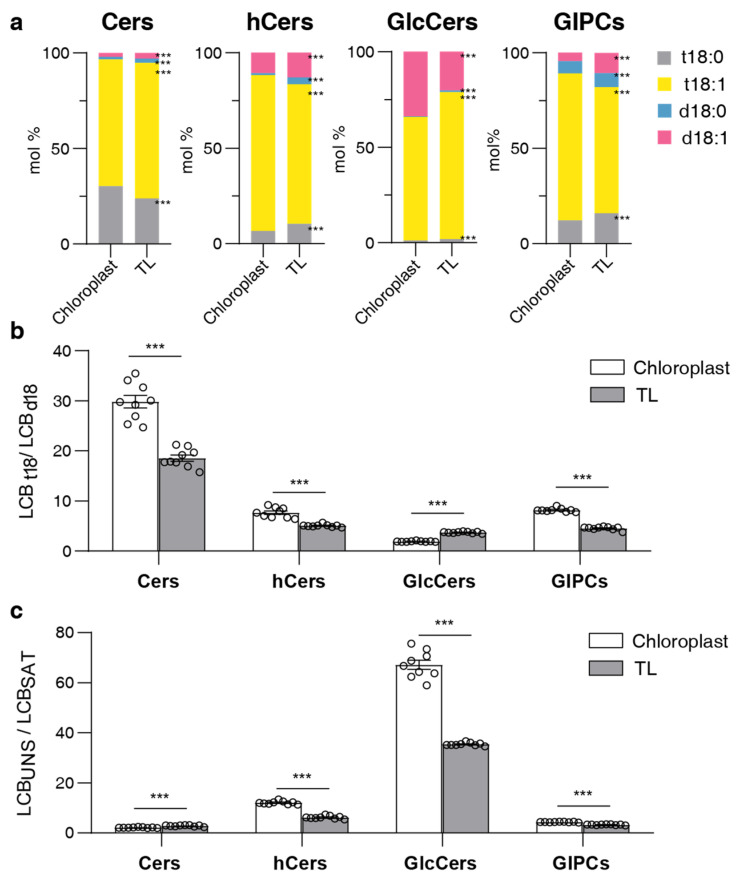
LCB composition in different sphingolipid classes. (**a**) LCB composition of four sphingolipid classes in chloroplasts and total leaves (TL). Bars of different colors represent four LCB species (t18:0, t18:1, d18:0, d18:1). Length of bars represents the average relative content (mol %) from nine replicates of each LCB species. (**b**) LCB_t18_/LCB_d1_8 ratio of each sphingolipid class; the ratio is calculated using trihydroxylated forms (t18:0 and t18:1) versus dihydroxylated forms (d18:0 and d18:1). (**c**) LCB_UNS_/LCB_SAT_ ratio of each sphingolipid class; the ratio is calculated using unsaturated LCB (t18:1 and d18:1) versus saturated LCB (t18:0 and d18:0). Error bars represent the means ± SE from nine individual plants. Different letters labeled on the top of bars indicate statistical differences using two-tailed Student’s t-tests (***: *p* < 0.001).

To assess the specific composition of sphingolipids in complex sphingolipids (Cers, hCers, GlcCers, GIPCs), we considered four LCBs (t18:0, t18:1, d18:0, d18:1) and ten FAs with lengths ranging from 16 to 26C (16:0, 18:0, 20:0, 20:1, 22:0, 22:1, 24:0, 24:1, 26:0, 26:1) which differ in length, degree of unsaturation, and C2-hydroxylation. For the LCB composition of complex sphingolipids, the predominant form was trihydroxylated (t18) and unsaturated forms in both the chloroplast and TL samples (Figure 4a,b). However, when considering this proportion, chloroplast samples differed substantially from the TL samples. Specifically, the proportion of t18 forms was higher in chloroplast samples than in TL for Cer, hCer, and GIPC classes but lower for the GlcCer class (Figure 4b). Concurrently, unsaturated forms were higher in chloroplast samples than in TL, especially in the GlcCer class (Figure 4c).

**Figure 5:**
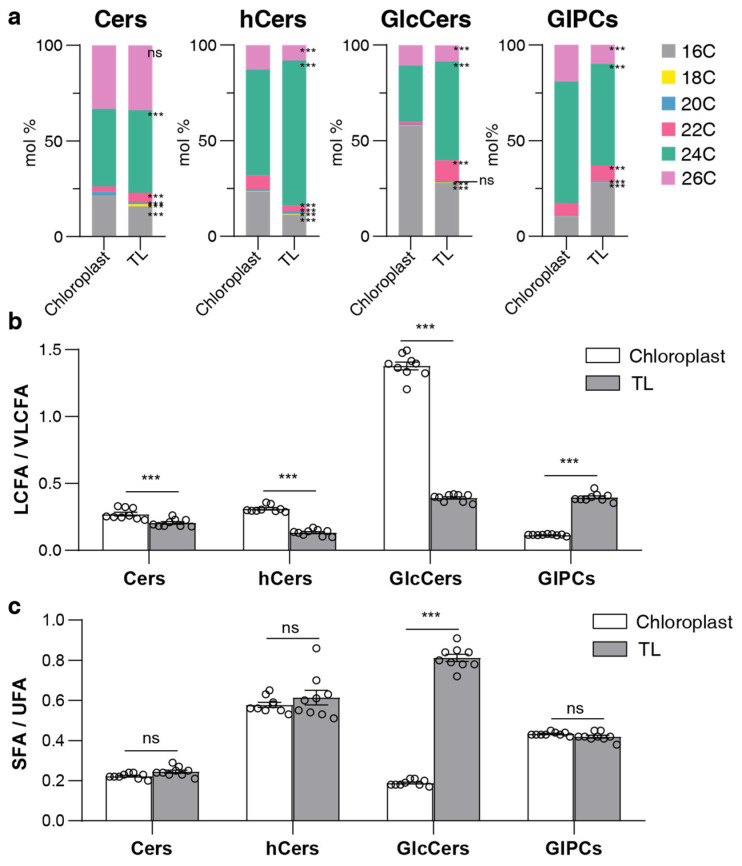
Fatty acid composition in sphingolipid classes. (**a**) Fatty acid (FA) composition of four sphingolipid classes; bars of different colors represent six FA species with 16–26C lengths (16C, 18C, 20C, 22C, 24C, 26C). The length of the bars represents the average relative content (mol %) from three biological replicates and three technical replicates of each FA species. (**b**) LCFA/VLCFA ratio of each sphingolipid class; the ratio is calculated using long-chain fatty acids (LCFA, 16C, and 18C) versus very long-chain fatty acids (VLCFA, 20C, 22C, 24C, and 26C). (**c**) SFA/UFA ratio of each sphingolipid class; the ratio is calculated using saturated fatty acids (SFA, 16:0, 18:0, 20:0, 22:0, 24:0, 26:0) versus unsaturated fatty acids (UFA, 20:1, 22:1, 24:1, 26:1). Error bars represent the means ± SE from nine individual plants. Different letters labeled on top of the bars indicate statistical differences using two-tailed Student’s *t*-tests (ns: *p* > 0.05, ***: *p* < 0.001).

In terms of the FA composition, all classes, with the exception of GlcCer in chloroplast samples, were predominantly composed of very long-chain fatty acids (VLCFAs) (20C, 22C, 24C, 26C) as opposed to long-chain fatty acids (LCFAs) (16C and 18C) (Figure 5b). Notably, Cer, hCer, and GlcCer in chloroplast samples were composed of a higher proportion of the 16C form than in TL, particularly GlcCer, which contained more than 50% of the 16C form and was the only sphingolipid class with an LCFA/VLCFA ratio greater than one (Figure 5b). Conversely, GIPC in chloroplasts contained a lower proportion of the 16C form, accompanied by a higher proportion of 24C and 26C forms than TL (Figure 5a,b). Calculating the ratio of unsaturated to saturated FA showed that all sphingolipid classes were dominated by unsaturated FA forms, but the proportion of saturated forms in GlcCer was significantly higher in chloroplast samples compared to TL (Figure 5c). Collectively, LCB and FA compositions exhibited notable differences between the chloroplast and TL samples, indicating that chloroplasts have a unique sphingolipid composition.

### 2.5. Predominant Sphingolipid Species

**Figure 6:**
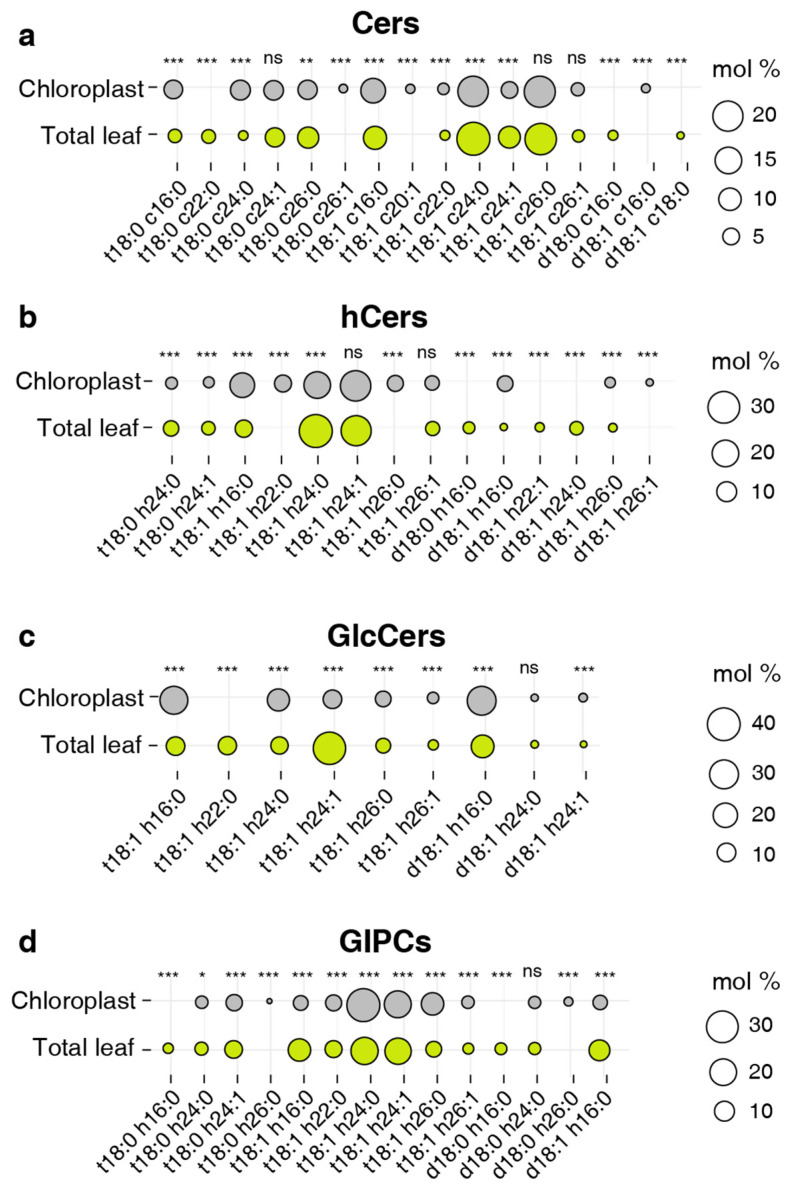
Distribution of abundant species from four sphingolipid classes. (**a**) Cers, (**b**) hCers, (**c**) GlcCers, and (**d**) GIPCs. The size of each bubble is related to the average relative content from nine individual plants for each individual species. Only sphingolipid species with an abundance of more than 1% for each sphingolipid class (16 species of Cers, 14 species of hCers, 9 species of GlcCers, and 14 species of GIPCs) are shown. Different letters labeled on the top of bars indicate statistical differences using two-tailed Student’s t-tests (ns: *p* > 0.05, *: *p* < 0.05, **: *p* < 0.01, ***: *p* < 0.001).

We detected a considerable number of sphingolipid species, but many of them were present in low abundance (Supplementary Table S2). To further scrutinize the potentially significant sphingolipid species, we classified those with a relative content exceeding 1% as abundant species. This classified a total of 49 species, including 16 species of Cers, 14 species of hCers, 9 species of GlcCers, and 14 species of GIPCs as abundant species (Figure 6).

Among the abundant Cer species, t18:1 c24:0 and t18:1 c26:0 were the most abundant in both chloroplast and TL samples, accounting for 40%~50% of the total (Figure 6a). For the hCer class, both sample types were overwhelmingly dominated by t18:1 h24:0 and t18:1 h24:1, constituting approximately 50% of the total, and a greater proportion of t18:1 h16:0 and d18:1 h16:0 was involved in the chloroplast sphingolipid assembly (Figure 6b). GIPC was also dominated by forms of t18:1 h24:0 and t18:1 h24:1, with the proportion of the t18:1 h24:0 form in chloroplasts being about 10% higher than that in TL (Figure 6d). Although the predominant species of the above three sphingolipid classes were consistent in chloroplasts and TL, the most abundant species of GlcCer showed a clear difference between the chloroplast and TL samples. In TL, the t18:1 h24:1 form was the predominant species, while in chloroplast samples, t18:0 h16:0 and d18:1 h16:0 forms were the predominant species (Figure 6c). This further underscores the specificity of chloroplast sphingolipid composition.

## 3. Discussion

This study presents the detailed identification of the sphingolipid composition of Arabidopsis chloroplasts. The sphingolipidome of the total leaves (TL) identified in our study aligns with those reported in previous studies [14,28]. When compared to other reported sphingolipidomes in plant tissues and cellular structures, the chloroplast exhibited commonalities and unique characteristics.

In terms of sphingolipid class distribution, GIPCs constituted the primary sphingolipid class in chloroplasts, accounting for approximately 60% of the total. This is similar to observations for the plasma membrane (PM), detergent-resistant membrane (DRM), microsomal membrane (MIC), endoplasmic reticulum (ER), nuclei, and mitochondria (Mito) [22,23,24]. GIPCs have been previously reported to serve as crucial membrane components in plants for pathogen recognition [29], as well as in response to environmental stresses such as elevated levels under cold stress and assisting in the influx of Ca^2+^ under salt stress [30,31]. The structural complexity and predominance of GIPCs could align with a wide variety of membrane functions, although the exact functional role and mechanism of GIPCs in chloroplasts remain unknown. Moreover, the proportion of GIPCs to the total sphingolipids in chloroplasts was lower than that in TL, while there was a more pronounced presence of free LCBs and hCer in the chloroplast.

Robust lipid trafficking between the chloroplast and ER occurs within the endomembrane system [32,33], suggesting that the accumulation of free LCBs in the chloroplast could function as a reservoir for sphingolipid biosynthesis. Notably, the LCB has been reported to be a potent signaling molecule that influences phytohormone levels and plays pivotal roles in programmed cell death [34,35], hinting at a potential relationship between the accumulation of LCBs in chloroplasts and the execution of these functions. As for hCer, their abundant presence in chloroplasts could be attributed to their hydrophobic properties. It has been reported that the stratum corneum necessitates the hydrophobicity of hCers to regulate water flow [36], and this property of hCers has been utilized to elucidate the accumulation of hCers in vacuolar membranes (VMs) [23], suggesting that the accumulation of hCers in chloroplasts may be driven by a similar mechanism.

For specific sphingolipid composition, the individual characteristics among TL and other cellular structures warrant further attention. To achieve this purpose, we compared the results from previous studies [22,23,24] with our results for a comprehensive discussion (Table 1 and Table 2). It is noteworthy that all reported sphingolipid data are derived from LC-MS/MS technology. To ensure consistency in their comparison, original data have been converted to relative content.

For Cer, the primary components remained consistent across the reported cellular structures, with t18:1 c24:0 and t18:1 c26:0 predominating, except for Mito, where d18:1 c24:1 was the most abundant component (Table 2). Additionally, a higher presence of 16C forms (primarily d18:1 c16:0) was observed in MIC, VM, chloroplast, and PM compared to other sample types (Table 1).

For hCer, t18:1 h24:0/1 were the predominant forms across each cellular structure and in the TL, with Mito also exhibiting a higher presence of d18:1 h24:0 (Table 2). Interestingly, d18:1 h24:0 also appeared as an abundant fraction in the TL (Figure 6a), suggesting that this species could be significantly contributed by Mito. Additionally, the nuclei were characterized by a substantial accumulation of sphingolipid with the t18:0 form and 26C form, distinguishing it from other structures (Table 1).

GlcCer and GIPC, as major plant sphingolipid classes, play a crucial role in maintaining membrane morphology and function, as reported in several studies [37,38]. For GlcCer, all cellular structures and TL were dominated by unsaturated LCB forms, accounting for up to about 98% (Table 1). Concurrently, the chloroplast, DRM, PM, and VM exhibited a significant presence of the C16 form GlcCer, as evidenced by the predominance of d18:1 h16:0 and t18:1 h16:0 (Table 2). Additionally, Mito was completely dominated by d18:1 h24:1 with a percentage of 66% (Table 1, [24]), and GlcCer with 24C and 26C forms accounted for more than 85% in the Mito and nuclei [22,24]. For GIPC, all cellular structures were dominated by the t18:1 24:0/1 forms of GIPC, and Mito contained almost exclusively 24C and 26C forms (Table 1).

Previous reports have indicated that very long-chain fatty acids (VLCFAs) (including 24C and 26C) are the primary sphingolipid components and play a crucial role in plant growth and development [39,40]. However, the results presented above suggest that internal cellular structures, such as the PM, chloroplast, and VM, exhibit a significant propensity to accumulate sphingolipids with 16C forms. Previous in vitro studies discovered that the connections between VLCFA ceramides exhibit stronger interactions, facilitating the establishment of stable and complex biological systems. In contrast, shorter 16C fatty acids are mostly phase-separated [41]. Therefore, the accumulation of the 16C form in the PM, chloroplast, and VM could potentially be associated with the motility of the endomembrane system and its intricate metabolic processes. Interestingly, the sphingolipid composition of Mito appears to be unusual in that it contains a large number of complex sphingolipids in the form of d18:1 and 24C, and sphingolipids with the d18:2 form and 28C form were identified in Mito [24], but not detected in the chloroplast or in other reported cellular structures. Historically, d18:2 has only been found in the pollen and flowers of Arabidopsis [42]. It remains unclear whether these components are related to the specific function of Mito. These findings further suggest that the distribution of sphingolipids is asymmetrical and uneven in plant cells, and whether this distinction in sphingolipid classes and species correlates with organelle-specific functions warrants further exploration.

Furthermore, it should be noted that in this study, the entire chloroplast was extracted without distinguishing specific structures, such as the inner membrane, outer membrane, thylakoids, and stroma. Therefore, the distribution of sphingolipids within each part of the chloroplast remains unknown.

In conclusion, our study pioneers the examination of the sphingolipidome of plant chloroplasts, thereby enhancing the sphingolipid profile of plant organelles and providing a robust reference for comprehending the spatial distribution and functional roles of sphingolipids across different cellular structures. Simultaneously, gaining insights into the sphingolipid composition of chloroplasts, which constitute a significant pool of organic carbon sources in plants, is of paramount importance for a profound understanding of chloroplast functions.

## 4. Materials and Methods

### 4.1. Plants and Growth Conditions

*Arabidopsis thaliana* wild-type (Columbia, Col-0) seeds were sterilized with chlorine for 1h and then spread on ½ of the MS medium. After 3 d of stratification at 4 °C, the seeds were grown at 22 °C under a 16 h light/8 h dark cycle with a 4800 to 6000 lux light intensity for 1 week before planting in a soil mixture (a mix of peat soil and vermiculite at 3:1, *v*:*v*). All the leaves were harvested at 4 weeks old. Some leaves were used for chloroplast isolation immediately, and others were lyophilized and then stored at −80 °C as total leaf samples for sphingolipid extraction. Three independent biological replicates from at least nine individual plants were collected.

### 4.2. Chloroplast Isolation

In order to isolate the chloroplasts, the protoplasts were prepared first. Four-week-old Arabidopsis leaves were harvested and chopped into a 20 mL enzyme solution (1.5% cellulase “Onozuka” R-10 (210824-01, Yakult, Tokyo, Japan), 0.4% macerozyme R-10 (110830-01, Yakult, Japan), 0.4 M of mannitol, 20 mM of KCl, 10 mM of CaCl_2_, 0.1% BSA and 20 mM of MES, pH 5.7). The leaves were gently shaken in the dark for 3 to 4 h until the protoplasts were released into the solution. The protoplasts were collected through a 40 μm filter and centrifugated at 800 rpm for 3 min.

The protoplasts were resuspended with chloroplasts in an isolation buffer (CIB, 1×) (0.3 M of sorbitol, 5 mM of MgCl_2_, 5 mM of EGTA, 5 mM of EDTA, 10 mM of NaHCO_3_, 20 Mm of HEPES, and pH 8.0) and chloroplasts were isolated with a Percoll gradient, as described previously, with minor modifications [43]. Briefly, two rounds of homogenization were completed by washing the precipitation on ice with a 1× CIB solution. The protoplasts were broken, and chloroplasts were released during the homogenization. We transferred the homogenate onto the top of the premade continuous Percoll gradient. To separate intact chloroplasts from broken chloroplasts and other debris, the homogenate-loaded Percoll gradient was centrifuged in a swinging-bucket rotor at 7800× *g* for 10 min with the brake off at 4 °C. The following two green bands were visible in the gradient: the lower green band contained intact chloroplasts, whereas the upper band contained broken chloroplasts. The lower green band was collected and washed using an HMS buffer (50 mM of HEPES, 3 mM of MgSO_4_, 0.3 M of sorbitol, and pH 8.0), then stored at −80 °C until further analysis. All experimental operations were performed on ice, using cut 1 mL pipette tips. The resulting chloroplasts of each biological replicate were equally divided into three parts as follows: one for protein quantification, one for sphingolipid (LCBs, Cers, hCers, and GlcCers) analysis, and one for GIPCs analysis.

### 4.3. Protein Quantification and Immunoblotting Assays

The total amount of protein extracted from the chloroplast and leaf samples was determined using the Coomassie Protein Assay Regent (WJ337105, Thermo Fisher Scientific, Waltham, MA, USA). BSA was employed as a standard. The purity of the isolated samples was detected via immunoblotting using organelle-specific marker antibodies as follows: BAK1 (plasma marker, AS12 1858, Agrisera AB, Vännäs, Sweden); BIP2 (ER marker, AS09 481, Agrisera AB, Sweden); RbcL (chloroplast marker, AS08 325, Agrisera AB, Sweden); V-ATPase (vacuole marker, AS07 213, Agrisera AB, Sweden); VADC1 (mitochondrionl marker, AS07 212, Agrisera AB, Sweden); Histone H3 (nuclear marker, AS10 710, Agrisera AB, Sweden). RbcL was also used as a loading control.

### 4.4. Sphingolipid Analysis

Samples for sphingolipid analysis were prepared, and the components of sphingolipids were determined using LC-MS/MS, as described previously [14,22,44]. Briefly, chloroplast samples or 300 mg of lyophilized leaf samples were homogenized. The internal standards (C17 base d-erythro-sphingosine and C12-Ceramide) were added and extracted with isopropanol/hexane/water (55:20:25, *v*/*v*/*v*). After incubation at 60 °C for 10 min, the supernatants were dried using nitrogen. For the detection of GIPCs, the dried extract was de-esterified by dissolving it in 2 mL of a 33% methylamine solution in ethanol/water (7:3, *v*/*v*), which was incubated at 50 °C for 1 h. After being dried with nitrogen, the samples were dissolved in 200 μL of methanol. Samples were analyzed using a 2.0 mm × 150 mm Luna 3 µm C8 column (Phenomenex, Torrance, CA, USA).

For LCBs, Cers, hCers, and GlcCers, sphingolipid detection was performed using liquid chromatography (AB SCIEX ExionLC AD, Sciex, Framingham, MA, USA) coupled with a triple quadrupole mass spectrometer (AB SCIEX 4500, Linthicum, MD, USA). Fractions containing sphingolipid were infused into the Turbo V™ ion source with a temperature of 450 °C, a curtain gas of 25, gas1 at 55 and gas2 at 55, a needle voltage of +5000 V, and a collision gas of 5.

For GIPCs, detection was performed using liquid chromatography (Agilent 1200SL, Agilent, Santa Clara, CA, USA) coupled with a triple quadrupole mass spectrometer (Agilent 6410, USA). The eluate from the column was directed to the ESI source. The probe was charged with 5000 V. The temperature was held at 350 °C, the gas flow was set at 10 L/min, and the nebulizer was 25 psi. Raw data were collected and processed using the SCIEX OS version 1.7.0 and Agilent Masshunter version 10.1 quantitative analysis software.

### 4.5. Data Analysis

Partial least squares–discriminant analysis (PLS-DA) was constructed using PlantMetSuite [45]. The heatmaps and bubble plots of sphingolipid components were performed using R 3.6.3 packages “pheatmap” and “gglot2”. Two-tailed Student’s t-tests were used to evaluate statistically significant differences between the two groups (* *p* < 0.05, ** *p* < 0.01, *** *p* < 0.001).

## Figures and Tables

**Table 1:** Sphingolipid composition of cellular structures in *Arabidopsis thaliana*.

	Cer	hCer	GlcCer	GIPC	Tissue	Ref.
TL	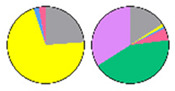	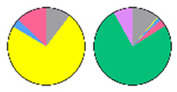	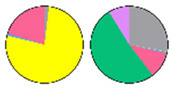	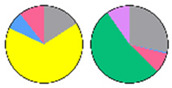	Leaf	This study
MIC	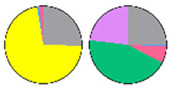	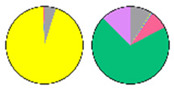	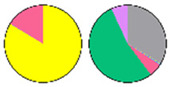	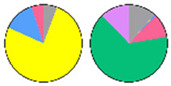	Leaf	[23]
DRM	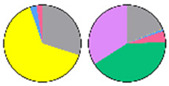	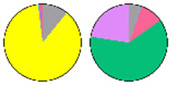	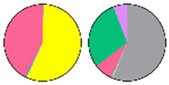	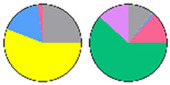		
PM	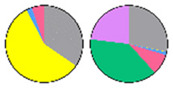	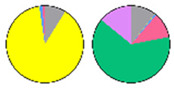	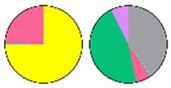	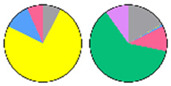		
VM	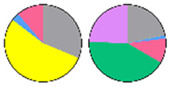	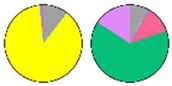	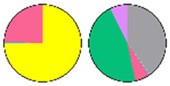	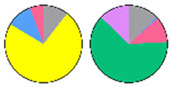		
Mito	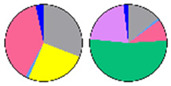	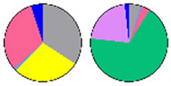	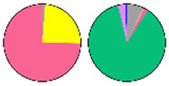	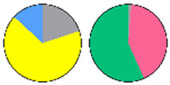	Leaf	[24]
Chloroplast	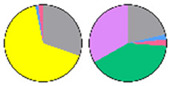	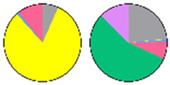	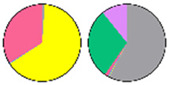	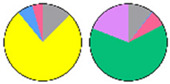	Leaf	This study
ER	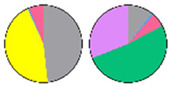	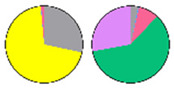	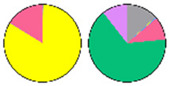	**/**	Seedling	[22]
Nuclei	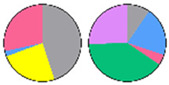	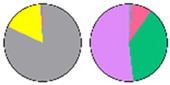	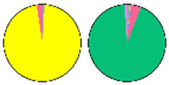	**/**		
LCB ■t18:1 ■t18:0 ■d18:0 ■d18:1 ■d18:2 FA ■16C ■18C ■20C ■22C ■24C ■26C ■28C						

TL: Total leaf, MIC: microsomal membrane, DRM: detergent-resistant membrane, PM: plasma membrane, VM: vacuolar membranes, Mito: mitochondria, ER: endoplasmic reticulum, LCB: long chain base, FA: fatty acid.

**Table 2:** Predominant sphingolipid species of cellular structures in *Arabidopsis thaliana*.

	Cer	hCer	GlcCer	GIPC	Tissue	Ref.
TL	t18:1 c24:0 t18:1 c26:0	t18:1 h24:0 t18:1 h24:1	t18:1 h24:1	t18:1 h24:0 t18:1 h24:1	Leaf	This study
MIC	t18:1 c24:0 t18:1 c26:0 t18:1 c16:0 t18:1 c24:1	t18:1 h24:1 t18:1 h24:0	t18:1 h24:1	t18:1 h24:0 t18:1 h24:1	Leaf	[23]
DRM	t18:1 c24:0 t18:1 c26:0	t18:1 h24:0 t18:1 h26:0 t18:1 h24:1	d18:1 h16:0	t18:1 h24:0 t18:0 h24:0		
PM	t18:1 c24:0 t18:1 c26:0 t18:0 c16:0	t18:1 h24:1 t18:1 h24:0	t18:1 h24:1 d18:1 h16:0 t18:1 h16:0	t18:1 h24:1 t18:1 h24:0		
VM	t18:1 c24:0 t18:1 c26:0 t18:0 c24:0	t18:1 h24:1 t18:1 h24:0	t18:1 h24:1 d18:1 h16:0 t18:1 h16:0	t18:1 h24:1 t18:1 h24:0		
Mito	d18:1 c24:0 t18:1 c26:0	t18:0 h24:0 d18:1 h24:0	d18:1 h24:1	t18:1 h24:1 t18:1 h24:0	Leaf	[24]
Chloroplast	t18:1 c24:0 t18:1 c26:0	t18:1 h24:1 t18:1 h24:0 t18:1 h16:0	d18:1 h16:0 t18:1 h16:0	t18:1 h24:0 t18:1 h24:1	Leaf	This study
ER	t18:0 c24:0 t18:1 c24:0 t18:1 c26:0	t18:0 h24:0 t18:1 h24:0 t18:0 h26:1	t18:1 h24:0 t18:1 h24:1	/	Seedling	[22]
Nuclei	t18:0 c24:0 d18:1 c20:0	t18:0 h26:1 t18:0 h24:0	t18:1 h24:1	/		

TL: Total leaf, MIC: microsomal membrane, DRM: detergent-resistant membrane, PM: plasma membrane, VM: vacuolar membranes, Mito: mitochondria, ER: endoplasmic reticulum.

## Data Availability

All pertinent data can be located in the article or Supplementary Tables. All other data in support of the results of this study are available upon reasonable request to the corresponding author.

## Supplementary Materials

The following supporting information can be downloaded at: https://www.mdpi.com/article/10.3390/plants13020299/s1, Supplementary Table S1: Absolute content of the sphingolipid profile of 160 sphingolipid species identified in TL and chloroplast samples. Supplementary Table S2: Relative content of the sphingolipid profile of 160 sphingolipid species identified in TL and chloroplast samples.
